# Number order in addition affects cognitive effort: evidence from mental arithmetic tasks

**DOI:** 10.3389/fpsyg.2025.1618197

**Published:** 2025-08-13

**Authors:** Omid Khatin-Zadeh, Danyal Farsani, Zahra Eskandari, Arash Ghahraman, Jiayong He, Hassan Banaruee

**Affiliations:** ^1^School of Foreign Languages, University of Electronic Science and Technology of China, Chengdu, China; ^2^Department of Teacher Education, Norwegian University of Science and Technology, Trondheim, Norway; ^3^Department of Theoretical Physics, Institute of Physics, Eötvös Loránd University, Budapest, Hungary; ^4^School of Foreign Languages, Tongji University, Shanghai, China; ^5^Department of Educational Psychology, University of Education Weingarten, Weingarten, Germany

**Keywords:** addition, arithmetic operation, order of numbers, processing, cognitive effort

## Abstract

In this study, our aim was to find out how order of numbers in the arithmetic operation of addition affects cognitive effort of mental processing. We presented two sets of addition questions (*a* + *b*) to a group of participants. In one set of questions, the first number of each item was larger than the second number (a > *b*). In another set of questions, the first number was smaller than the second number (a < *b*). The participants were asked to answer each item within a period of 12 seconds. The results showed that when the first number was larger than the second number, participants provided more correct answers and were faster in giving correct answers. Two explanations are discussed for these results. Finally, it is concluded that the property of commutativity of addition does not mean that performing that operation in various situations involves the same level of cognitive effort.

## 1 Introduction

Numerical cognition and mental processes involved in arithmetic operations have been the subject of a large body of research in recent years (Ashkenazi, 2024; Ashkenazi and Adi, 2023; Bender and Beller, 2011; Fischer et al., 2012; Jiang et al., 2024; Khatin-Zadeh et al., 2025; Xu et al., 2019; Yu et al., 2023). Number and arithmetic operations are fundamental concepts in mathematics. Therefore, it is crucially important for mathematics educators to know how numbers and arithmetic operations are represented and processed in the mind. In this area of research, one line of research has focused on how numbers and arithmetic operations are embodied. In mathematics textbooks, numbers are represented and embodied in terms of fixed points on a straight line that has direction, and the arithmetic operations of addition and subtraction are represented in terms of rightward and leftward movements along this line (Khatin-Zadeh et al., 2024). In this description, the addition of a positive number to another number is described as a rightward movement, and the addition of a negative number to another number is described as a leftward movement. Conversely, the subtraction of a number by a positive number is described as a leftward movement, and the subtraction of a number by a negative number is described as a rightward movement. Based on the strong version of embodied cognition (Gallese and Lakoff, 2005), numbers and arithmetic operations of addition and subtractions are embodied as points and movements along a straight line (Lakoff and Núñez, 2000). From this perspective, sensorimotor systems are actively involved in the processing of numbers and arithmetic operations. Importantly, the motor system, which guides human body movements, plays a partial role in the processing of arithmetic operations as these operations are represented and embodied in terms of movements.

Some behavioral and neuroimaging studies have provided evidence that supports the active role of body movements and sensorimotor systems in the processing of various mathematical concepts, such as geometry concepts (Smith et al., 2014; Walkington et al., 2024), numbers and arithmetic operations (e.g., Ansari and Dhital, 2006; Fischer and Shaki, 2018; Prado et al., 2011; Spitzer et al., 2014; Walkington et al., 2019). For example, results of two studies showed that people tended to point to the right after responding to an addition problem and to the left after responding to a subtraction problem (Pinhas and Fischer, 2008; Pinhas et al., 2014). Some findings showed that solving addition problems was followed by a shift of attention to the right, and solving subtraction problems was followed by a shift of attention to the left (Masson and Pesenti, 2014). Results of one study showed that making a rightward movement or looking rightward had a positive impact on the performance of students in responding to addition problems, while a leftward movement or looking leftward had a positive impact in responding to subtraction problems (Sixtus et al., 2023).

In addition to behavioral studies that have supported embodied nature of numbers and arithmetic operations, some neuroimaging studies have focused on the areas of brain and sensorimotor networks that are involved in the processing of numbers and arithmetic operations. Results of these studies have suggested that parietal cortex plays a crucial role in the processing of numbers and arithmetic operations (Park et al., 2013). Specifically, some studies have shown that bilateral intraparietal sulcus and areas around it are significantly activated when an individual performs number comparison tasks and arithmetic operations (hochon et al., 1999; Dehaene et al., 1999; Pinel et al., 2001; Ansari and Dhital, 2006; Prado et al., 2011). These studies have suggested that the process of perceiving magnitude is primarily handled by right parietal region, while arithmetic operations and symbolic comparisons, which involve more advanced and more precise number processing, are mainly conducted by both left and right parietal cortex. Not only independent processes conducted in left and right parietal cortex but also functional connectivity and interaction between these regions play a role in performing the arithmetic operations of addition and subtraction (Park et al., 2013). This means that various areas of brain are activate when an individual performs an arithmetic operation.

Based on the findings of mentioned studies, it can be assumed that mental processes involved in an arithmetic operation can be divided into a set of sub-processes and sub-features. For example, the arithmetic operation of addition (*a* + *b*) involves an initial process of perceiving magnitudes of *a* and *b*. This is followed by another process through which the quantity of *b* is added to the quantity of *a*. At each stage, various areas of brain and various mechanisms are employed to obtain the result of the operation. Also, the arithmetic operation of *a* + *b* has some features. For example, *a* and *b* can be small or very large numbers. The cognitive effort of mentally calculating the result of *4* + *15* is lower than the cognitive effort of mentally calculating the result of 37 + 48. Therefore, when an individual is asked to mentally calculate the result of *a* + *b* (without using paper, pencil, or any other tool), the magnitudes of *a* and *b* is one of the features that affects level of cognitive effort of mentally calculating the result of *a* + *b*. Being commutative is another feature of the arithmetic operation of addition. Being commutative means that the result of *a* + *b* is equal to the result of *b* + *a*. A question that is raised here is that whether changing the order of *a* and *b* changes the mental effort of obtaining the result of *a* + *b*. One of the authors of this paper has had the experience of teaching and learning mathematics in five different countries (Iran, UK, Brazil, Chile, and Norway). In his teaching experiences, he observed that in performing the arithmetic operation of *a* + *b*, students were faster and more accurate in answering when *a* was bigger than *b*. This was particularly the case with dyscalculic students and those who required more educational attention. However, before conducting this study, he had not tested this in an experimental setting. Some past studies have investigated relationship between numerical order processing and arithmetic abilities (e.g., Lyons and Ansari, 2015; Lyons et al., 2014; Vogel et al., 2017, 2015). However, to the best of our knowledge, no study has examined the impact of order of numbers on mental processes involved in arithmetic operations.

In this paper, we define *cognitive effort* for performing a cognitive task on the basis of two criteria: (1) degree of accuracy of people in providing correct answer for that task; (2) the amount of time that people need to provide correct answer for that task. If people provide more correct answers in shorter periods of time to a cognitive task, we assume that cognitive effort for performing that task is lower. Level of cognitive effort for performing a task is in fact its level of difficulty. We hypothesized that changing the order of *a* and *b* affects mental processes and the effort of processing when an individual tries to obtain the result by mental calculation. To test this hypothesis, we asked a group of participants answer a set of addition problems in two sessions. In one session, 30 addition problems (*a* + *b*) were given to the participants. They were asked to mentally calculate the result of each item. Then, order of *a* and *b* was changed in each item, and 30 new items were created. These new addition problems were given to the participants in another session. The aim was to compare the performance of participants in calculating the results of additions when the second number was larger than the first number with the performance of participants in calculating the results of additions when the second number was smaller than the first number. In other words, the basis of this comparison was accuracy in providing correct answers and reaction time for providing correct answers. We wanted to learn whether order of the addends in adding two numbers influences: (a) the tendency to answer correctly, (b) the reaction time of providing correct answer.

## 2 Method

### 2.1. Participants

Participants in this study consisted of 30 foreign language learners, all native Persian speakers, with an age range of 17 to 34 years (*M* = 25.5, SD = 5.5). The sample included 18 females and 12 males, ensuring a balanced gender distribution. The sample size for this study was determined based on a preliminary power analysis using data from the first 10 participants. Specifically, a paired *t*-test was conducted, yielding a statistically significant effect (*t* = −12.97, *p* < 0.001), indicating a strong effect size. Given this robust statistical outcome, the results suggested that a sample size of 30 participants would be sufficient to maintain adequate power while ensuring reliability. Furthermore, previous studies utilizing the same research approach have employed comparable sample sizes, reinforcing the appropriateness of this choice. Participants were randomly selected from a larger pool, ensuring representative distribution. The same group took part in both sessions of the experiment. Participation was voluntary, with all individuals providing written informed consent. The study was conducted in accordance with the Declaration of Helsinki (World Medical Association, 2013).

### 2.2 Materials

Two sets of addition problems were used in this study. Each set included 15 addition problems (totally 30 items). Each item appeared only once for the participants. This prevented any effects of test familiarity on the correctness of the answers and the speed of answering the items. Each item in one set of problems had a reversed item in the other set. For example, if (23 + 68) belonged to one set, (68 + 23) belonged to the other set. In each item, participants had to mentally calculate the result of an addition (*a* + *b*). In one set of questions, *a* was larger than *b*; in another set, *a* was smaller than *b*. The addends were two-digit numbers. In all questions, the right digit of the larger number was larger than the right digit of the smaller number. In four items, both addends were even; in four items, both addends were odd; in 22 items, one addend was odd and one addend was even. The list of the items has been given in the Appendix. These two sets of questions were scrambled and presented to the participants randomly in one session. A 0.9 s fixed interval was designed between each two addition questions followed by a non-relevant question, such as “what is your favorite color” (see the Appendix). Therefore, the experiment included 30 addition items, 30 unrelated questions to control the speed of the participants between each loop of trials, and 30 intervals to spare time for cognitive processing of the participants. In each item, the problem appeared on the screen of a computer for a maximum of 12 s. During this time, participants had to answer the item by pressing keys on the keyboard of the computer. Then, the next question appeared on the screen.

### 2.3 Procedure

Before conducting the experiment, participants attended a training session. In this session, the details of the experiment were explained for the participants, and several items were presented to them to make them familiar with the questions. In the main experiment of the study, each participant sat on a chair in front of the screen of a computer. To ensure that the participants were fully ready and familiar with the questions, detailed oral instructions were again given to them. As mentioned, in each item, an addition problem in the form of *a* + *b* appeared on the screen of the computer for a period of 12 s. During this time, participants had to type the answer in a box by using keyboard of the computer. The answers and time of answering each item were recorded for data analysis.

### 2.4 Data analysis

As mentioned, among the 30 addition problems that were in the form of *a* + *b*, in one set of 15 items, the first number (a) was larger than the second number (b); in the other set of 15 items, the first number was smaller than the second number. In the first stage of data analysis, the total number of correct answers for each set of items was obtained for each participant. For example, one of the participants provided 11 correct answers for the first set of questions (items in which a >*b*) and 7 correct answers for the second set of items (items in which a < *b*). Then, the total number of correct answers for each set was obtained for all participants.

In the second stage of data analysis, for each item of addition problems, the time of providing a correct answer was recorded in an excel sheet. This was done for each set of question in two separate columns of an excel sheet. Then, an unpaired *t*-test was used to compare the mean time of providing correct answers for the first set of items (items in which a >*b*) with the mean time of providing correct answers for the second set of items (items in which a < *b*).

### 2.5 Summary of the experimental protocol

In this study, participants completed a computerized mental addition task consisting of two distinct sets of arithmetic problems, each comprising 15 unique items (30 items in total). Each problem was presented only once per participant to prevent familiarity effects. The two sets were structurally equivalent. In one set, the first addend was larger than the second (a > b, e.g., 39 + 18); in the other, the first was smaller (a < b, e.g., 15 + 37). All addends were two-digit numbers, without controlled parity distribution across the sets. The problems were randomized and interleaved with neutral, unrelated filler questions (e.g., What is your favorite color?) to minimize strategy adaptation and standardize pacing. Each trial began with a 0.9 s inter-trial interval, followed by the presentation of an addition problem on the computer screen for up to 12 s. During this time, participants were instructed to mentally calculate the sum and type their response using a keyboard. The filler question then followed, completing the trial. Prior to the main experiment, participants underwent a training session that included instruction and practice items to ensure familiarity with the task. During the experiment, each participant completed 30 trials in a single session, with both accuracy and response times automatically recorded. For data analysis, the total number of correct responses was calculated separately for each condition (a > b vs. a < b), and mean response times for correctly answered items were computed. Accuracy was analyzed using a mixed-effects logistic regression model with condition as a fixed effect and participant as a random intercept to account for subject-level variability. Response times were compared using a paired-samples *t*-test to assess whether the order of operands influenced the speed of correct mental calculation.

## 3 Results

The total number of correct answers for each set of questions was obtained. Among the 450 answers (30 participants × 15 items) provided for the first set of questions (items where the first number was larger), 281 answers were correct. For the second set (items where the second number was larger), 221 answers were correct.

To account for subject-level variability, we compared the accuracy between the two sets of questions using a paired statistical test (a mixed-effects logistic regression model with subject as a random effect). This analysis showed that the first set of questions resulted in significantly more correct answers than the second set (*p* < 05). A paired *t*-test was used to make a comparison between the mean time of providing correct answers for the first set of questions with the mean time of providing correct answers for the second set of questions. The *p-value* of this test was smaller than 0.001 (*t* = 13.5275).

A binary logistic regression was conducted to examine the effect of condition on the likelihood of producing a correct response. The model was statistically significant, χ^2^(1) = 3.99, *p* = 0.046, indicating that condition significantly predicted response accuracy. Specifically, compared to the condition with second larger number condition (reference category), participants in the first larger number condition were significantly more likely to respond correctly (*B* = 0.27, *SE* = 0.134, *t* = 2.003, *p* = 0.046). The odds of a correct response were 30.8% higher in the First Larger condition [OR = 1.308, 95% CI (1.005, 1.702)] (Tables 1, 2).

**Table 1:** Binary logistic regression predicting correct responses from condition (*N* = 900).

**Predictor**	***B***	**SE**	***t***	**Sig**.	**95% CI (B)**	**Exp (B)**	**95% CI for Exp (B)**
Intercept	−0.30	0.095	−3.097	0.002	[−0.48, −0.11]	0.744	[0.617, 0.898]
Condition: first larger	0.27	0.134	2.003	0.046	[0.005, 0.532]	1.308	[1.005, 1.702]
Condition: second larger	(ref)						

Reference category for condition is second larger number Exp (B) = Odds ratio.

**Table 2:** Full results of the *t*-test for comparing the times of providing correct answers for the two sets of questions.

**Question**	**First set of question (a > b)**	**Second set of questions (a < b)**
Mean	6.416	7.158
SD	0.632	0.590
SEM	0.037	0.039
Number of correct answers	281	221

**Figure 1:**
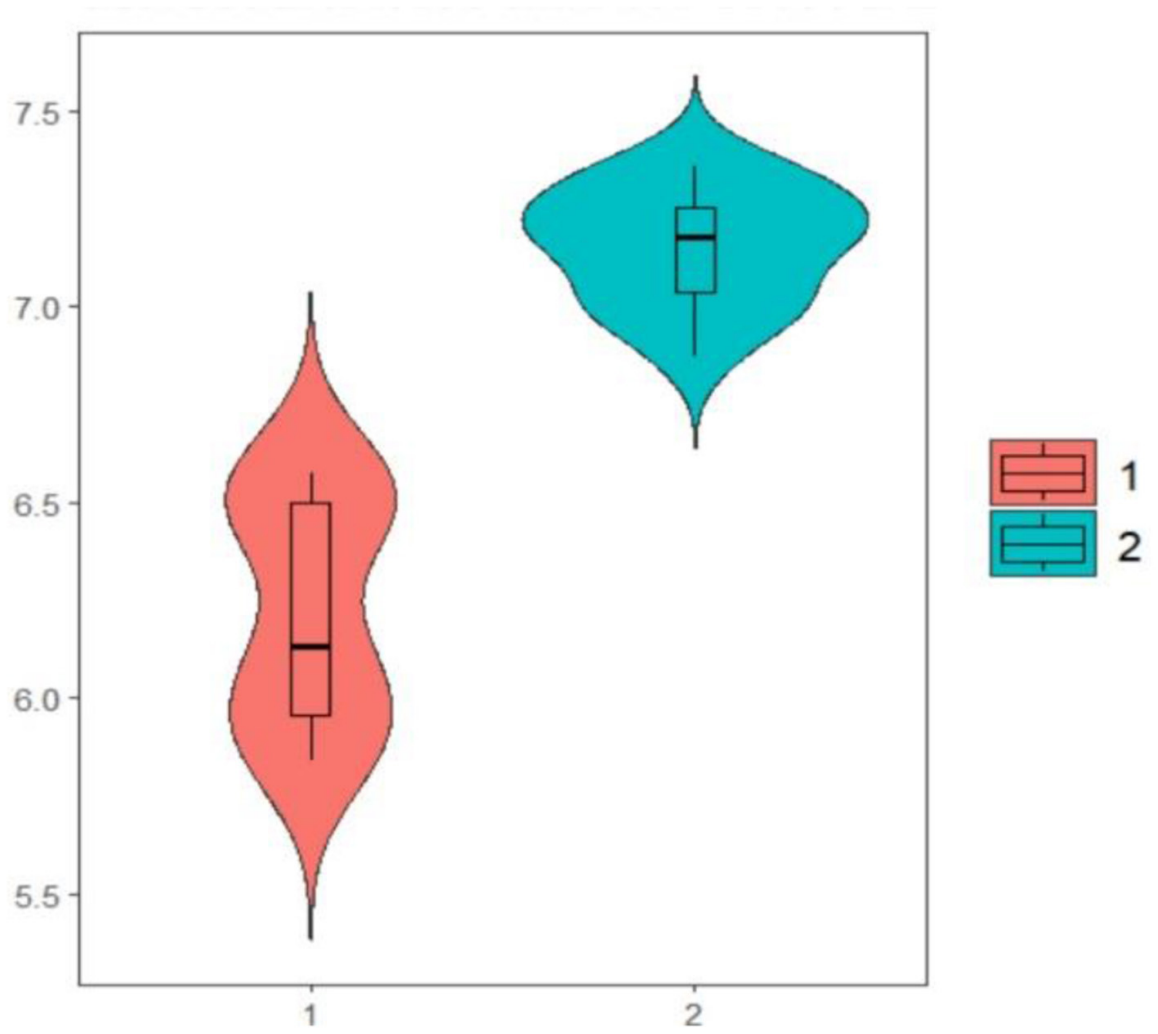
Mean time of providing correct answers for the two sets of questions.

Figure 1 shows a visual description of mean times of providing correct answers. In this figure, the times for providing correct answers has been shown on the vertical axis. It can be seen that the times for providing correct answers for the first set of items (shown by 1) is lower than the times for providing correct answers for the second set of items (shown by 2).

The results presented in the table and figure show that the mean time of providing correct answers for the first set of questions was significantly shorter than the mean time of providing correct answers for the second set of questions.

## 4 Discussion

As mentioned in the previous section, results of our study showed that order of numbers in an addition operation had a significant impact on the performance of participants in answering the questions. Specifically, adding a smaller number to a larger number was easier and less effortful than adding the larger number to the smaller number. For example, obtaining the result of *37*+*15* was easier than obtaining the result of *15*+*37*. This suggests that mental operations involved in obtaining the result of *15*+*37* are more complex and more demanding than mental operations involved in obtaining the result of *37*+*15*. A question that is raised here is that why obtaining the result of *15*+*37* is more difficult than obtaining the result of *37*+*15*. Since the arithmetic operation of addition is commutative, results of these two operations are the same. Why should obtaining the result of the first operation be more demanding and more effortful than the second operation? Here, we discuss two explanations for this observation; one is based on the findings of behavioral studies and another one is based on neuroimaging evidence.

### 4.1 Behavioral explanation

The effect of order on the difficulty of an addition problem can be explained on the basis of behavioral studies on embodied numerical cognition. As mentioned, results of some past studies have shown that arithmetic operation of addition (*a* + *b*) is embodied as a rightward movement along a horizontal line (Pinhas and Fischer, 2008; Pinhas et al., 2014) or an upward movement along a vertical line (e.g., Wiemers et al., 2014). This line is an axis that has direction. From this perspective, any addition operation involves three elements: a starting point, a rightward/upward movement along a straight horizontal/vertical line, and an ending point. The starting point is the position of the first number (*a*) on the axis. The length of the rightward/upward movement is equal to the value of the second number. The ending point is the result of the addition (*a* + *b*). This suggests that mental operations that are involved in the processing of the first number (*a*) are different from mental operations that are involved in the processing of the second number (*b*). The first number in the addition is represented as a fixed point on a straight line, while the second number is represented as a rightward/upward movement along the axis. This means that the second number is simulated in terms of a rightward fictive motion on a straight line, as suggested by Pinhas and Fischer (2008) and Pinhas et al. (2014)). This indicates that the way the first number is processed is different from the way that the second number is processed. Because of this difference, sensorimotor resources that are employed to represent, embody, and process the first number are different from sensorimotor resources that are recruited to represent and process the second number. Specifically, the motor system is actively employed to process the second number as it is represented as a rightward/upward movement along a horizontal/vertical line (Khatin-Zadeh and Yazdani-Fazlabadi, 2023). This is supported by the findings of studies showing that processing sentences referring to a fictive motion involves a mental simulation of the fictive motion (Matlock, 2004, 2010) and also studies that have found evidence suggesting that an area of the motor system that responds to visually-perceived motions is involved in the processing of fictive motion (e.g., Saygin et al., 2010; Marghetis and Núñez, 2013). Based on these past findings, it can be suggested that the peak of effort of mental processing for calculating *a* + *b* starts when *b* is presented to the individual, because at this point the motor system enters the stage as a supporting cognitive resource. When *b* is larger, this stage is more demanding, and more cognitive effort is needed to obtain the result of *a* + *b*. It is more difficult because a larger fictive movement should be handled by the motor system and other resources that are involved in the mental operations. In other words, level of difficulty of obtaining the result of *a* + *b* is dependent on how high is the peak of processing effort. If *b* is larger, the peak of effort for processing (the moment that *b* is presented) is higher and the whole process of obtaining the result of operation is more demanding and effortful.

A question that may be raised here is that the arithmetic operation of addition may not always be simulated in terms of a movement on a straight line. One may add the single digits and obtain the final result without such a mental simulation. Why should mental simulation of a larger fictive motion affect cognitive effort needed to obtain the result of the arithmetic operation? To answer this question, it should be noted that even when people do not consciously simulate an addition in terms of movement on a straight line, this simulation can be activated in the mind. This can happen because at elementary levels of learning arithmetic operations people learn this arithmetic operation by simulating it in terms of movement on a straight axis. This association between this arithmetic operation and movement on a straight line is stored in the memory and can be activated even at higher levels of mathematics (even years later). This association is in fact the embodied memory for the arithmetic operation of addition.

### 4.2 Neuroimaging explanation

As mentioned, results of some past studies have shown that mental processes and areas of brain that are involved in basic magnitude processing differ from mental processes and areas of brain that are involved in performing arithmetic operations such as addition and subtraction (e.g., Park et al., 2013). These studies have provided evidence suggesting that basic quantity processing is mainly handled by right parietal region of brain, while precise number processing such as arithmetic operations and symbolic comparison tasks are handled by both left and right parietal cortex (Ansari and Dhital, 2006; hochon et al., 1999; Dehaene et al., 1999; Pinel et al., 2001; Prado et al., 2011; Price and Ansari, 2011). Furthermore, as mentioned, results of a study conducted by Park et al. (2013) have suggested that functional connectivity and interaction between right and left parietal cortex play a critical role in representing numbers and performing arithmetic operations. This means that obtaining the result of the operation *a* + *b* involves both right and left parietal cortex as well as an interaction between these two areas of brain.

In the process of obtaining the result of *a* + *b*, the quantity of *a* and then the quantity of *b* are processed in the mind of the induvial. Based on the findings of past studies (Ansari and Dhital, 2006; hochon et al., 1999; Dehaene et al., 1999; Pinel et al., 2001; Prado et al., 2011; Price and Ansari, 2011), it can be said that at this stage, right parietal cortex is probably the only area that is activated to prepare the ground for obtaining the result of the arithmetic operation. At the next stage, the quantity of *b* is added to the quantity of *a*. Based on the findings of past studies [studies reviewed in Park et al. (2013)], it can be said that at this point, left parietal cortex comes into play. Results of our study suggested that order of numbers in the arithmetic operation affects the interaction between right and left parietal cortex. When *a* is presented to the individual, it is kept in the short-term memory. This quantity is taken as the base or the starting point of mental processes for obtaining the result of the arithmetic operation. Presenting *b* to the individual is immediately followed by adding *b to a*. This stage involves an active process conducted primarily on *b*. The quantity of *a* is taken as the base or starting point, and the mental process for obtaining the result of arithmetic operation is mainly focused on *b*. Results of our study suggested that complexity or difficulty of this stage is dependent on the quantity of *b*. That is, if the quantity of *b* is larger, the process conducted in this stage is more difficult and more demanding. This means that degree of difficulty or complexity of an addition operation is mainly dependent on the quantity of the second number in addition operation. In other words, the activation of left parietal cortex and interactions that take place between right and left parietal cortex are largely dependent on the second number in an addition problem. In other words, although the process of addition involves the quantities of two numbers and the mental calculation of addition, it is the quantity of the second number that determines how complex the process is. While the processing of the first number is primarily handled by right parietal cortex, the processing of the second number and the operation that is performed on it is handled by both right and left parietal cortex. In other words, in obtaining the result of *a* + *b*, the areas of the brain that are involved in the processing of *a* are different from the areas that are involved in the processing of *b*. When *b* is larger, a larger part of the brain should handle and process a larger quantity of information. This involves a more demanding level of processing. That can explain why degree of difficulty of an addition is affected by the order of numbers.

## 5 Conclusion

Based on the results of this study, it can be concluded that two very similar arithmetic operations may involve different levels of mental processing. Although being commutative (*a* + *b* = *b* + *a*) is one of the fundamental properties of arithmetic operation of addition, levels of cognitive efforts involved in calculating the results of *a* + *b* and *b* + *a* are not the same. Level of difficulty varies according to which one of the two numbers is larger. Therefore, when a mathematical operation is represented in terms of abstract symbols, the property of commutativity of that operation does not mean that performing that operation in various situations involves the same level of cognitive effort. In the case of addition, level of cognitive effort is dependent on the magnitudes of numbers involved in the operation (obtaining the result of *23* + *49* is more difficult than obtaining the result of *12* + *6*), order of numbers (obtaining the result of *8* + *39* is more difficult than obtaining the result of *39* + *8*), and possibly some other factors.

Results of our study can have some practical implications for mathematics education. Students come to school with diverse mathematical, linguistic, cultural, social, and emotional experiences that influence how they approach and understand mathematics. This is particularly the case with elementary levels of mathematics education. Teaching practices that are adapted to students' experiences and preferences can prevent them from experiencing mathematics as difficult and help them overcome potential mathematical difficulties. However, to date, little research related to preferred and inclusive mathematical practices has been conducted in countries that researchers of this study has been working in (Iran, UK, Brazil, Chile, and Norway). For example, there is a noticeable gap of research conducted under the Norwegian National Curriculum (LK20) (Opplæringslova, 1998), particularly research conducted on students with different mathematical abilities and dyscalculic students. The Norwegian school is based on inclusion in the sense that students attend their local school, and the school is to adapt to the students' diverse needs (Opplæringslova “The Norwegian Education Act,” 1998). Therefore, in this study, we explored how aspects of something as basic as the preferred arithmetic positioning operations can be used as a preferred pedagogical tool empowering students with different mathematical learning abilities, particularly lower attainers in mathematics. Empowerment for participation is also emphasized in the New Norwegian Education Act (Opplæringslova, 1998) and in the UN Convention on the Rights of the Child, Article 12 (World Conference on Special Needs Education, 1994). Finally, it should be noted that there are many mathematical operations and relations that have the property of commutativity. Examining cognitive efforts that are involved in the processing of these operations and relations in various conditions is a question that can be investigated in future studies.

## Data Availability

The original contributions presented in the study are included in the article/Supplementary material, further inquiries can be directed to the corresponding author.

## Appendix

### List of questions used in this study:

15+37
37+15
23+28
28+23
64+96
96+64
24+79
79+24
26+77
77+26
44+97
97+44
18+39
39+18
57+68
68+57
88+56
56+88
45+87
87+45
63+88
88+63
24+75
75+24
33+66
66+33
15+48
48+15
45+68
68+45

### List of non-relevant questions

1. What is your favorite color?
2. What is your favorite weather?
3. What country were you born in?
4. Which season do you like most?
5. Are you interested in reading news?
6. Are you interested in sports?
7. What is your favorite hobby?
8. What is your favorite food?
9. Are you interested in music?
10. Are you interested in politics?
11. How many languages can you speak?
12. Can you drive a car?
13. What is your favorite sport?
14. Are you interested in reading novels?
15. Can you ride a bicycle?
16. Can you swim?
17. Are you interested in visiting museums?
18. Do you have any brother?
19. Do you like to live in a village?
20. Have you visited a foreign country?
21. Are you a sociable person?
22. Are you interested in watching movies?
23. What is the capital of your country?
24. What province do you live in?
25. Are you interested in exercising?
26. Are you interested in going to restaurants?
27. What country would you like to visit in future?
28. Do you go to cinemas?
29. Do you like to travel with a train?
30. Are you interesting in visiting rural areas?
